# Cluster of Angiostrongyliasis Cases Following Consumption of Raw Monitor Lizard in the Lao People’s Democratic Republic and Review of the Literature

**DOI:** 10.3390/tropicalmed6030107

**Published:** 2021-06-22

**Authors:** Leeyounjera Yang, Chirapha Darasavath, Ko Chang, Vilayvanh Vilay, Amphonesavanh Sengduangphachanh, Aphaphone Adsamouth, Manivanh Vongsouvath, Valy Keolouangkhot, Matthew T. Robinson

**Affiliations:** 1 Adult Infectious Disease Ward, Mahosot Hospital, Vientiane 01000, Laos; chonje2016@gmail.com (L.Y.); chiraphadarasavath@gmail.com (C.D.); me.chang2009@gmail.com (K.C.); valy.keoluangkhot@gmail.com (V.K.); 2 Infectious Disease Ward, 103 Military Hospital, Vientiane 01000, Laos; vvilayvanh64@gmail.com; 3 Microbiology Laboratory, Mahosot Hospital, Vientiane 01000, Laos; Amphone@tropmedres.ac (A.S.); Manivanh@tropmedres.ac (M.V.); 4 Lao-Oxford-Mahosot Hospital-Wellcome Trust Research Unit (LOMWRU), Mahosot Hospital, Vientiane 01000, Laos; Aphaphone.A@tropmedres.ac; 5 Centre for Tropical Medicine & Global Health, Nuffield Department of Medicine, University of Oxford, Oxford OX3 7LG, UK

**Keywords:** angiostrongyliasis, *Angiostrongylus cantonensis*, eosinophilic meningitis, Laos, zoonosis

## Abstract

Angiostrongyliasis in humans causes a range of symptoms from mild headache and myalgia to neurological complications, coma and death. Infection is caused by the consumption of raw or undercooked intermediate or paratenic hosts infected with *Angiostrongylus cantonensis* or via contaminated vegetables or water. We describe a cluster of cases involved in the shared meal of wild raw monitor lizard in the Lao PDR. Seven males, aged 22–36 years, reported headaches, abdominal pain, arthralgia, myalgia, nausea/vomiting, diarrhea, neurological effects and loss of appetite. Five were admitted to hospital. The final diagnosis was made by clinical presentation and case history, and positive *A. cantonensis* PCR for two cases. All hospitalized patients recovered fully following supportive treatment. The remaining two individuals sought local home remedies and made full recovery. Whilst most published reports concern infections via consumption of molluscs, few detailed reports exist on infections that result from the consumption of reptiles and there exists little awareness in Lao PDR. This case cluster, which originates from a single meal, highlights the potential public health risk of the consumption of raw and wild-caught meat in Lao PDR and the Southeast Asia region. Without specific diagnostics, clinical history and the consideration of recent food consumption are important when evaluating patients.

## 1. Introduction

*Angiostrongylus cantonensis*, also known as the rat lungworm, is a nematode responsible for human angiostrongyliasis. It is an emerging zoonotic pathogen and, while it is endemic in East Asia and Southeast Asia, it has become established globally. By 2012, over 2904 cases had been recorded globally [1,2]. In Hawai’i cases have steadily climbed, with an average of 2.4 cases/year between 2010 and 2014 increasing to 9 cases in 2015, and 21 cases occurred in 2016 [3]. In China, outbreaks involving between 8 and 160 individuals [4] have occurred in addition to sporadic cases and seroprevalence studies suggest a prevalence of 0.8% sero-positivity in the general population, which raises to 7.4% in those directly involved in aquaculture or the processing of snails [5]. In Lao People’s Democratic Republic (Lao PDR), *A. cantonensis* has been identified in cases of eosinophilic meningitis (EM), with 11% of 35 patients possessing >10% eosinophils in cerebrospinal fluid (CSF) obtained by diagnostic lumbar puncture, and being qPCR positive for *A. cantonensis* [6].

The life cycle of *A. cantonensis* is well documented [1,2,7] and cycles through rats, who are the definitive hosts, and molluscs (notably slugs and snails), which are intermediate hosts. Humans become infected by consuming the intermediate or paratenic hosts or via water or vegetation contaminated by larvae [8]. In humans, the worms are unable to complete the lifecycle and remain in the CNS. While the worms may remain in either the subarachnoid spaces or meninges, further migration may occur in the brain, including migration to the eye. Damage resulting from migration and the death of the worms themselves can result in an inflammatory response and the development of a meningo-encephalitis syndrome [9]. This is typified by a CSF eosinophilia constituting >10% of the total CSF leukocyte count [6].

The history (consumption of host species) usually suggests the diagnosis in a patient with compatible symptoms. Visualization of the worms in CSF is the gold standard, but it is rare. The majority of patients present with headache (95%); neck stiffness (46%), paresthesia (44%), vomiting (38%) and nausea (28%) are also reported [1]. In addition, neurological symptoms include face or limb paralysis, photophobia and diplopia (resulting from migration of the worms to the eye). In severe cases, continuous high intracranial pressure may occur (from the inflammatory response) and may result in coma and death [10,11]. Treatment is usually supportive.

**Figure 1:**
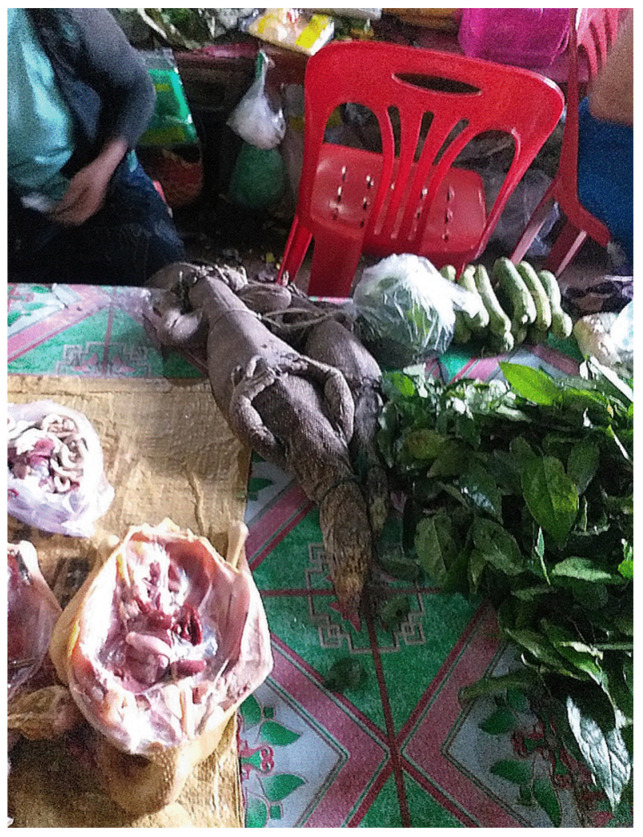
Live monitor lizard sold at a local wet market in the Lao PDR.

Infection of *A. cantonensis* is typically associated with the consumption of an undercooked or uncooked (raw) host species, predominantly molluscs, but also frogs, centipedes, crustaceans, fish and lizards [6,12,13,14,15]. Consumption of raw protein dishes is common in the Lao PDR and not only include fish [16] but also snails (in a dish called ‘koi hoi’) and lizard. Monitor lizards can routinely be observed being sold at wet markets in the Lao PDR (Figure 1), with estimates putting typical yearly trade at 4536 individual lizards per market [17,18]. Monitor lizards of the genus *Varanus* have been well documented as paratenic hosts for *A. cantonensis*. Yellow tree monitors (*V. bengalensis*) from across Thailand have been shown to be infected, with 95.5% of those sampled positive for *A. cantonensis* [19].

We describe a cluster of angiostrongyliasis cases in seven individuals related to the consumption of a single meal of raw monitor lizard meat. In addition, we review all the reported cases of angiostrongyliasis following consumption of reptile meat.

## 2. Case Presentations

**Figure 2:**
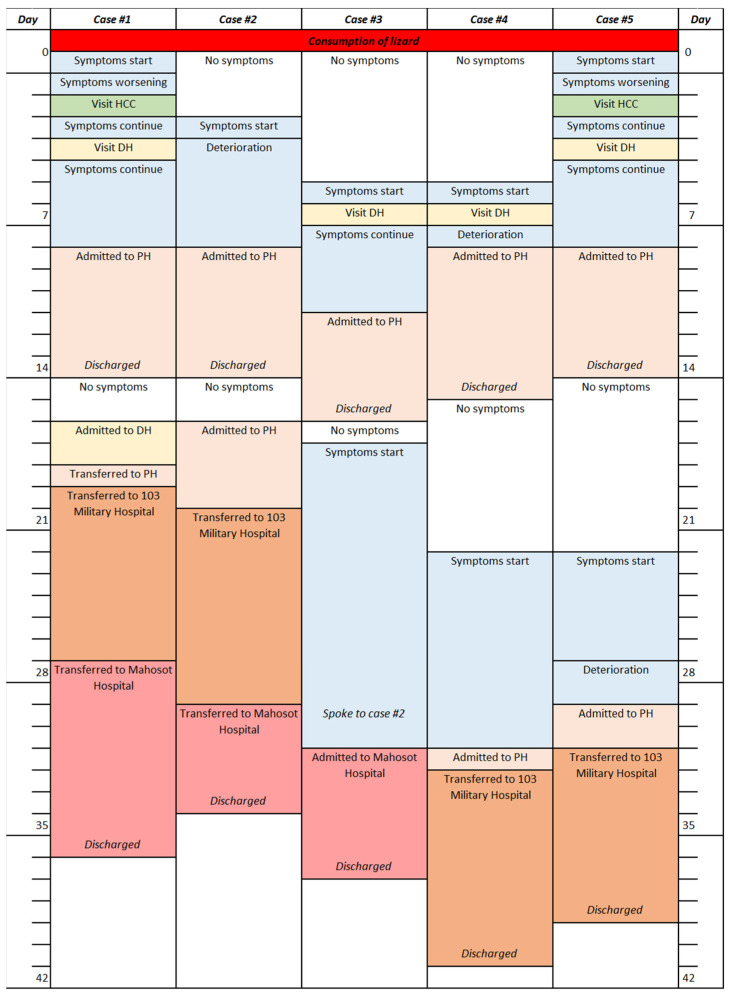
Timeline for cases 1 to 5. HCC = Healthcare clinic; DH = District Hospital; PH = Provincial Hospital.

Seven males, aged between 22 to 36 years, caught a wild monitor lizard and consumed the raw meat together in a shared meal in Vientiane Province, Lao PDR, on 21 May 2020. All seven individuals developed symptoms within one week. Case history for cases 1 to 5 is provided in Figure 2. Over a course of one month, cases 1 to 5 presented at either a local Healthcare Clinic and/or District Hospital. Cases 1 to 5 were later admitted to or transferred to the Provincial Hospital, 103 Military Hospital (Vientiane capital) and Mahosot Hospital (Vientiane capital). Initial treatments consisted of antibiotics and antipyretics, while no steroids were given. All five cases were successfully treated at Mahosot and 103 Military Hospitals following treatment with dexamethasone/prednisolone and albendazole and were discharged on days 34 to 41. Cases 6 and 7 did not attend any medical facilities and followed home treatments. Of the seven individuals, two were local farmers, while five were serving members of the Lao military. Individual case details are given below and in Table 1.

### 2.1. Case 1

A 22 year old male presented with severe headache, fever (39 °C), body pain, arthralgia and abdominal pain for a duration of one month. Initial symptoms were nausea, vomiting and watery diarrhea, for which on-set was approximately 1–2 h after the consumption of the lizard. Upon examination, there were no signs of meningitis, papilledema or focal neurological deficits. Computed tomography (CT) scan was normal but lumbar puncture (LP) CSF showed 515 cells/mm^3^ WBC (56% eosinophils, 44% lymphocytes). Admission diagnosis was meningitis and rickettsiosis. CSF was culture negative, along with Ziehl–Neelson and India Ink stains. Routine PCR analysis was carried out at Mahosot Hospital. PCRs for Dengue virus, *Orientia tsutsugamushi*, *Rickettsia* spp. and *Leptospira* spp. were all negative. *Angiostrongylus cantonensis* ITS1 qPCR [6,20] was performed on CSF and was positive (Cq = 35.24). Treatment was dexamethasone 8 mg IV twice per day for nine days until clinical recovery, followed by prednisolone administered orally at 1 mg/kg/day for a further five days (all steroid treatments were for a total of 14 days); albendazole 400 mg was administered twice daily for 14 days. Following commencement of treatment, a full recovery was made within nine days when the patient returned home (day 36) and completed treatment.

### 2.2. Case 2

A 25 year old male presented with severe headache, arthralgia, abdominal pain, dizziness, fatigue and loss of appetite for a one month period. Initial symptoms were body pain, arthralgia and fatigue, which appeared four days after consuming the raw flesh of the lizard. Admission diagnosis was meningitis. Upon examination he had papilledema, hence LP was not performed. CT-scan was normal. Following treatment with albendazole and steroids as per case 1, improvements were seen after three days, at which point the patient returned home (day 34) and switched to oral prednisolone.

### 2.3. Case 3

A 24 year old male presented with headache, body pain, arthralgia and focal neurological deficits (including numbness of the right arm and leg) for a one month period. Initial symptoms of headache, fatigue, loss of appetite and body pain appeared seven days after consuming the lizard. Admission diagnosis was meningitis. CT-scan was normal but CSF WBC was 90 cells/mm^3^ (66% eosinophils, 23% neutrophils and 11% lymphocytes). CSF was culture negative, along with Ziehl–Neelson and India Ink stains. PCRs were negative for Dengue virus, *O. tsutsugamushi*, *Rickettsia* spp. and *Leptospira* spp. *Angiostrongylus cantonensis* qPCR on CSF was positive (Cq = 30.70). The patient recovered fully after six days of treatment with steroid and albendazole as per case 1 and returned home (day 37), completing steroid treatment with oral prednisolone.

### 2.4. Case 4

A 24 year old male presented with severe headache, muscle weakness and numbness on arms and legs for a one month period. Initial symptoms of headache, nausea, vomiting and generalized body pain occurred seven days after the consumption of the lizard. Other symptoms included body pain, arthralgia and diplopia (no further eye examination was performed). The patient responded well to seven days of therapy with albendazole and steroids, as per case 1. The patient switched to oral prednisolone (day 41) and fully recovered seven days later.

### 2.5. Case 5

A 31 year old male presented with nausea, vomiting and arthralgia, which started 1–2 h after consuming raw lizard. Symptoms persisted for 1 month with severe headache, body pain, abdominal pain, fatigue and numbness of the fingers and toes. Upon examination, the patient was confused and had difficulty concentrating, but demonstrated no meningeal signs. No LP was performed. The patient improved and became completely asymptomatic within seven days of treatment with albendazole and steroids, as per case 1, and returned home (day 39) and continued the remaining course of treatment with oral prednisolone.

### 2.6. Cases 6 and 7

Limited information was available for cases 6 (36 year old male) and 7 (32 year old male). It was confirmed by telephone interview that case 6 had headache, body pain and arthralgia for a one week period after the consumption of the raw lizard. He recovered after one week following traditional herbal remedies and home treatment. Case 7 had nausea and vomiting, which started 1–2 h after consuming the raw lizard. Symptoms progressed to headache, body pain and arthralgia. He improved and recovered after 1 to 2 weeks following traditional herbal remedies and home treatment. Neither individual attended any medical facilities and no definitive diagnosis was made.

## 3. Discussion

While individual infections and large-scale outbreaks (over 100 patients being infected in China [21]) of angiostrongyliasis have been reported extensively in the literature, these reports result predominantly from the consumption of infected snails. Clinical infection through the consumption of lizard meat is less documented in the scientific literature, but it is well known through anecdotal evidence in endemic regions, particularly, Southeast Asia. A search in the literature up to September 2020 identified 36 cases of angiostrongyliasis with direct links to lizard consumption [22,23,24,25,26,27,28,29,30], although case details were only available for 20 of those.

Of the reported cases with clinical information, patients were aged between 15 to 64 years of age and 95% were males (19/20). Sixteen cases were reported from India [23,25,26,27,28], three from Thailand [22] and one from Sri Lanka [24]. While all patients were reported to have consumed monitor lizard (*V. bengalensis* when a species was noted), 35% specifically reported eating raw liver (7/20) and 60% reported eating raw flesh (12/20). Symptoms started from 0 days to 14 days after the consumption of lizard meat, with the initial symptoms being headache and/or vomiting. Symptoms persisted from 10 days to 6 weeks before hospital admission. All patients reported headaches (20/20), 80% (16/20) had fever, seven reported vomiting (35%) and six cases noted arthralgia, myalgia or abdominal pain. Sixty-five percent had neurological signs, including neck stiffness (11/11), papilledema (2/6), hyperreflexia (2/2) and muscle spasms (1/1). Five patients were reported as having hyperaesthesia and one with paraesthesia. One patient had seizures and was admitted in a coma. Thirteen had CT head scans of which twelve were reported as ‘normal’ whilst one was ‘unremarkable’. This last case also had an electroencephalogram (EEG), which suggested increased intracranial pressure; the CSF WCC, originally 384/mm^3^, increased to 560/mm^3^ with 50% eosinophils. Five cases had Magnetic Resonance Imaging (MRI) scans. All five patients with MRI scans showed varying degrees of periventricular intensities, lesions and hemorrhage. In one case, a migratory track and cavity could be observed.

Where conducted, blood work gave a white cell count (WCC) between 7210 to 16,200 cells/µL and eosinophils between 16 to 35%. Eight patients were reported to have ‘raised’ or ‘high’ CSF pressure (only one recorded, at 330 mm H_2_O), WCC 95 to 1200 cells/µL, eosinophils between 28 to 70% (or were reported as ‘eosinophilia’ or ‘severe EM’) and lymphocytes between 29 to 70%. CSF protein was between 75 to 249 mg/dL, while glucose was 40 to 92 mg/dL. In the two cases where blood glucose is also reported, CFS/plasma glucose ratios were 0.61 and 0.67. In one case, larvae could be seen in the CSF wet mount. Where stated, treatment predominantly consisted of steroids (13/16), with or without albendazole (7/16). In one case, symptoms worsened after albendazole, which was stopped after 24 h [24]. All of the 20 reported cases recovered. Diagnosis of all cases was primarily based on symptoms and clinical presentations, brain imaging and (in one case) visualization of worms in the CSF. None of the cases were confirmed by polymerase chain reaction (PCR).

Of the 16 cases without details, all were from Thailand [22,29,30]. Available information indicates that 14 recovered, one recovered but had paralysis and one case was fatal.

Interestingly, only two cases from the 20 with clinical information were directly linked: a 51 year old father and his 15 year old son ate raw liver following the recommendation of an indigenous healer to increase strength. Both were later admitted to the hospital and the father was in a coma while the son, who ate less, presented with the standard symptoms including body pain, headache and fever [26]. Of the 16 cases without details, six of those were potentially from two independent events, although precise details could not be confirmed (Jitpimolmard et al., 1992, in Kanpittaya et al. [22]).

A number of traditional dishes in Lao PDR use raw protein as their main ingredient, either in the form of flesh, specific organs or blood and this includes a number of known hosts and paratenic hosts for *A. cantonensis* (such as molluscs and lizard). While *A. cantonensis* has not been isolated in Lao PDR, initial studies using PCR [6] and anecdotal evidence from local clinicians would support the occurrence of angiostrongyliasis. This case series details the recorded incident of a cluster of *A. cantonensis* infections in the Lao PDR following a single meal of a monitor lizard (likely *V. bengalensis*). The symptoms seen across all seven individuals are highly classical of *A. cantonensis* infections. While mild to severe headaches are reported amongst most patients [1], this case series also reported some less frequent symptoms including fatigue, abdominal pain, muscle weakness and diarrhea. One patient (case 4) presented with diplopia. Diplopia has previously been reported in patients where larvae have been identified within the eye [31] and therefore suggests ocular angiostrongyliasis.

Although very similar symptoms were seen across all seven patients, their degree of intensity varied greatly, with some being mild enough for the patient to decide not to attend the hospital but to seek traditional herbal remedies. This would suggest that cases of angiostrongyliasis are likely to go unrecorded in the Lao PDR, suggesting an underestimation of the burden of the disease in the country. When the patients did attend hospital, a positive diagnosis was not made and appropriate supportive treatment was not given until almost one month after the consumption of the lizard and the onset of symptoms. This highlights the likelihood of under reporting, even with more serious presenting symptoms, and the need to build a wider awareness of the pathogen within clinicians in the Lao PDR.

Although positive qPCR diagnosis was only made in two of the seven cases, a confident diagnosis can be made based on clinical presentation and case history of lizard consumption as per previous reports described in the literature where none were diagnosed by PCR. Advanced diagnostic techniques are not essential at low-level healthcare centers in low-resource settings provided that appropriate case history is taken and recorded and clinicians are aware of such pathogens in their differential diagnosis.

As mentioned previously, treatment is usually supportive. Therapeutic lumbar punctures are often used to relieve pressure in the CNS and steroids given to reduce inflammation in the brain [24,32,33]. Steroids are often used in combination with anthelmintics to kill surviving worms and speed up recovery, although in some cases treatments with anthelmintics such as albendazole or thiabendazole have been seen to induce negative effects on the patients, most likely due to dead worms initiating further inflammatory responses [24]. Therefore, there is insufficient evidence for the most appropriate treatment plan.

## 4. Conclusions

While this is not the first case of angiostrongyliasis in the country (unpublished re-ports), this cluster of cases originating from a single meal highlights the presence of *A. cantonensis* in the Lao PDR and its potential to cause disease. In addition, this case cluster provides a unique detailed case history. Clinicians need to take this disease into account when assessing patients and, while specific diagnostics may not be available in much of the country, the consideration of clinical history and recent food consumption is important when evaluating patients.

## Figures and Tables

**Table 1:** Symptoms and clinical description of cases 1 to 5.

Case Number	1	2	3	4	5
Patient description					
Age, sex	22, M	25, M	24, M	25, M	31, M
Days (hours) after lizard consumption until onset of symptoms	0 (1–2 h)	4	7	7	0 (1–2 h)
Duration of illness (days)	36	32	32	36	39
Symptoms					
Headache	Y	Y	Y	Y	Y
Fever (°C)	Y (39 °C)	N	N	N	N
Abdominal pain	Y	Y	N	N	Y
Arthralgia	Y	Y	Y	N	Y
Myalgia/body pain	Y	Y	Y	Y	Y
Nausea/vomiting	Y	N	N	Y	Y
Diarrhea	Y	N	N	N	N
Loss of appetite	N	Y	Y	N	N
Neurological	N	Y	Y	Y	Y
Description of neurological symptoms	n/a	Fatigue, dizziness, papilledema	Fatigue, paraesthesiae	Diplopia, paraesthesiae, muscle weakness	Confusion, stupor, paraesthesiae
Blood					
WCC (cells/µL)	11,900	8420	6700	7500	8640
Neutrophils (%)	75.9	80.8	75.9	61.0	76.0
Lymphocytes (%)	20.5	17.2	18.2	27.0	19.0
Eosinophils (%)	32.0	0.5	0.4	4.0	0.0
Glucose (mg/dL)	76.1	99.1	79.2	101.0	258.0
CSF					
Lumbar puncture	Y	N	Y	N	N
Opening pressure (mm H_2_O)	25		15		
Lactate (CC)	20		20		
WCC (cells/µL)	515		90		
Neutrophils (%)	0.0		23.0		
Lymphocytes (%)	44.0		11.0		
Eosinophils (%)	56.0		66.0		
Protein (mg/dL)	Albu: 108		Albu: 126		
Glucose (mg/dL)	57		39		
Days to recovery from treatment start	9	3	6	7	7

## Data Availability

All available data is published in this manuscript.
